# Supplementary figures and images for: Clinical and molecular characterization of COVID-19 hospitalized patients
Source: PLoS One. 2020 Nov 18;15(11):e0242534. doi: 10.1371/journal.pone.0242534 (PMC7673557; doi:10.1371/journal.pone.0242534)

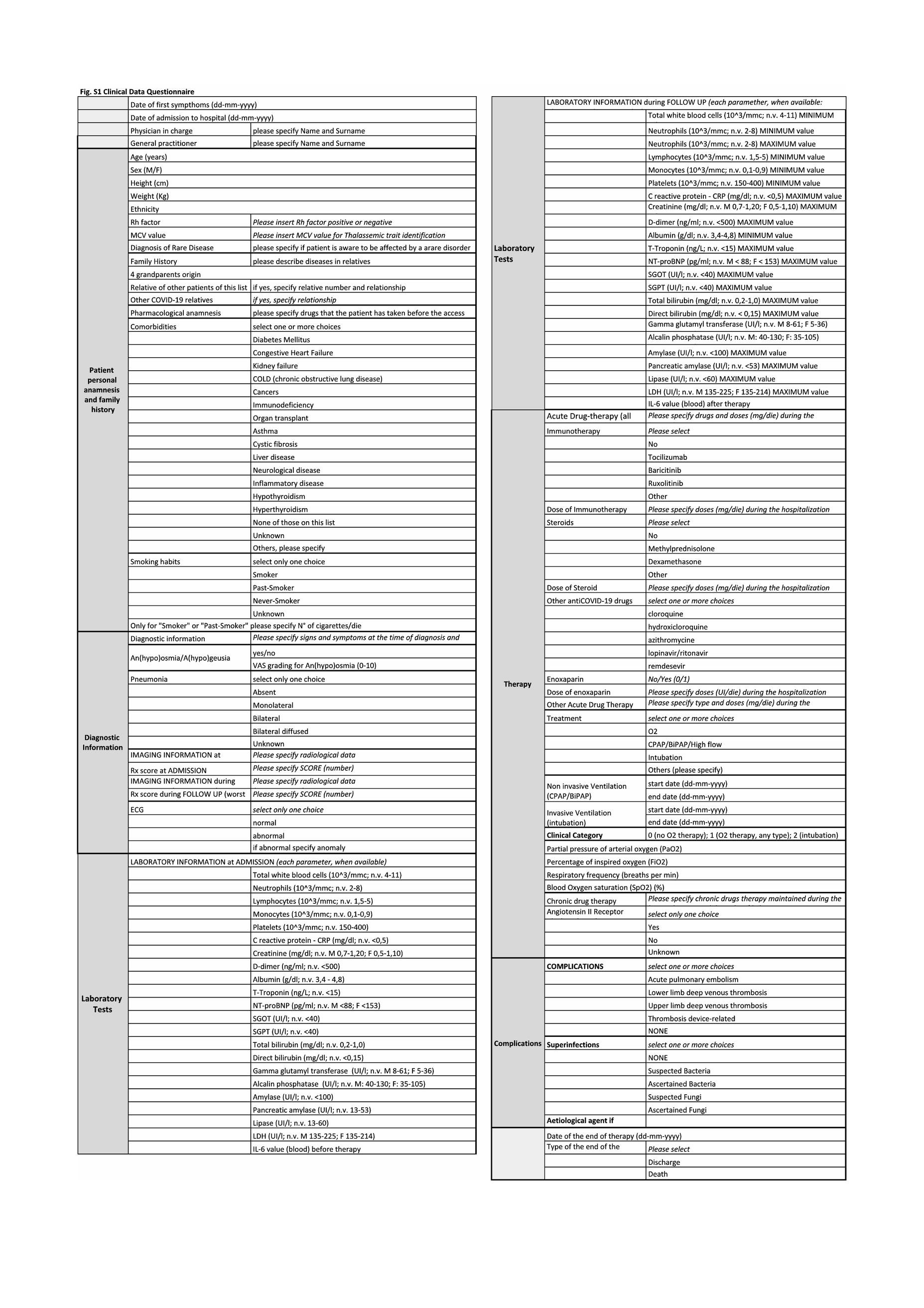

Supplement: S1 Fig — The Questionnaire includes five different categories of data: Patient personal anamnesis and family history, Diagnostic Information, Laboratory Tests, Therapy and Complications. Clinical data were collected in detail for all COVID-19 patients. (TIF) [file pone.0242534.s001.tif]
